# Advancing Care for Family Caregivers of persons with dementia through caregiver and community partnerships

**DOI:** 10.1186/s40900-018-0084-4

**Published:** 2018-01-22

**Authors:** Carole L. White, Kristen J. Overbaugh, Carolyn E. Z. Pickering, Bridgett Piernik-Yoder, Debbie James, Darpan I. Patel, Frank Puga, Lark Ford, James Cleveland

**Affiliations:** 1 School of Nursing, UT Health San Antonio, 7703 Floyd Curl Drive, San Antonio, TX 78229 USA; 2 School of Health Professions, UT Health San Antonio, 7703 Floyd Curl Drive, San Antonio, TX 78229 USA

## Abstract

**Background:**

There are currently 15 million Americans who provide over 80% of the care required by their family members with Alzheimer’s disease and other dementias. Yet care for caregivers continues to be fragmented and few evidence-based interventions have been translated into routine clinical care and therefore remain inaccessible to most family caregivers. To address this gap, the Caring for the Caregiver program is being developed at UT Health San Antonio, School of Nursing to improve support services and health outcomes for family caregivers. Our purpose is to describe the engagement process undertaken to assess caregiver and community needs and how findings are informing program development.

**Methods:**

We are using a model of public engagement that consists of communication of information, collection of information from stakeholders, and collaboration where stakeholders are partners in an exchange of information to guide program activities. An assessment of the community was undertaken to identify resources/services for family caregivers. Subsequently, stakeholders were invited to a community-academic forum to discuss strategies to build on existing strengths for family caregiving and to identify gaps in care. Detailed notes were taken and all discussions were recorded and transcribed for analysis. Data were analyzed using thematic content analysis.

**Results:**

We conducted site visits with 15 community agencies, interviewed 13 family caregivers, and attended community events including support groups and health and senior fairs. Fifty-three diverse stakeholders attended the community-academic forum. Participants identified existing assets within our community to support family caregivers. Consistent among groups was the need to increase awareness in our community about family caregivers. Themes identified from the discussion were: making the invisible visible, you don’t know what you don’t know, learning too late, and anticipating and preparing for the future.

**Conclusions:**

Incorporating caregiver and community stakeholders was critical to ensure that the priorities of our community are addressed in a culturally responsive accessible program for family caregivers. The forum served as important mechanism to partner with the community and will be an annual event where we can continue to work with our stakeholders around needs for practice, education, and research.

## Plain English summary

Family caregivers are the backbone of our healthcare system. They provide over 80% of the care needed by their family members with dementia but often struggle on their own with limited support. To address this need we are establishing a Caring for the Caregiver program. This has been undertaken in two stages. The first stage has focused on gathering information about the resources and services in our community to support family caregivers. The second phase involved bringing family caregivers, people from community agencies, healthcare professionals, and researchers from the university together in a forum to discuss priorities for addressing family caregiver needs. Discussions revealed that there was a need for a greater awareness in the community about the role that family caregivers take on to care for their loved ones with dementia. Several key themes for care were identified and included access to timely information and support through the progression of dementia and assistance with future care planning. Findings are informing education programs for family caregivers and also a program to facilitate advance care planning. Family caregivers and members of the community are working with the university researchers to plan the programs and also to design and conduct research to provide information on best practice for caregivers. This project shows the importance of working with family caregivers of those with dementia and those in the community to ensure that the programs we develop build on identified needs.

## Background

Alzheimer’s disease is the sixth leading cause of death in the United States and the only one among the top 10 without a means to prevent, cure or even significantly slow its progression. [1] The burden of Alzheimer’s disease and other forms of dementia extends far beyond the person with a diagnosis, with significant costs and consequences for the family, the healthcare system, and for society. Family caregivers (families, friends, neighbors) are especially impacted by the disease, not only related to the emotional impact of the diagnosis but also by the role they take on in care provision. Family caregivers provide over 80% of the assistance needed by their family members. [2] Caring for a family member with dementia can be particularly challenging because of the related cognitive decline as well as the changes in personality, behavior, and functional ability of the affected individual. Those providing care to persons with dementia are more likely to take on multiple responsibilities including assisting with activities of daily living, coordinating health care services, and managing finances as well as other instrumental activities of daily living compared with caregivers of persons without dementia. [3] Although some caregivers report positive benefits associated with the caregiving role such as a closer relationship with the care recipient or an opportunity to assist others, [4, 5] there is an extensive body of evidence showing the negative consequences on the caregiver’s physical, emotional, social, and financial health. [6–8]

Our current systems of support for caregivers are limited and fragmented with few evidence-based interventions translated into routine clinical care, thus remaining inaccessible to the majority of family caregivers. [9] Caregivers without support are much more likely to experience negative outcomes and transition out of the caregiving role. [10] Enabling family caregivers to continue providing care, while maintaining their own health and well-being, is a top priority in addressing Alzheimer’s disease and mitigating its impact on our health care system and on society.

To address the growing burden of dementia and its impact on the family, we are developing an evidence-based program, ‘Caring for the Caregiver’, to support family caregivers as part of an integrated care model for persons with dementia and their family caregivers. A priority in developing the caregiver program is to ensure cultural sensitivity in addressing the needs of our diverse community. Given the increased risk of Alzheimer’s disease and other dementias among Hispanics, [11] a program that is relevant to the needs of Hispanic caregivers is particularly important to our community which is over 50% Hispanic. Our goal is to establish a program that collaborates with community-based programs, building on the strengths and assets already existing within the community, to provide a coordinated program of support and resources for patients and families.

**Fig. 1:**
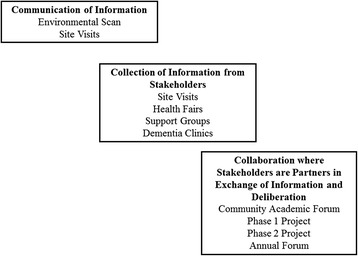
Model for caregiver and public involvement (based on Rowe and Fewer (15))

Patient and public involvement is increasingly considered essential to inform research and practice so that the perspectives of the community of interest are fully represented. [12] Evidence from a number of studies shows that engaging stakeholders in planning and decision-making contributes to more relevant programs and improves health care quality and outcomes. [13, 14] Rowe and Frewer [15] describe a spectrum of public engagement that includes communication of information, collection of information from stakeholders, and collaboration where stakeholders are partners in an exchange of information and deliberation. We used this framework as a guide for the activities undertaken to engage stakeholders in developing the Caring for the Caregiver Program (Fig. 1). The purpose of this paper is to describe the results of public engagement that led to the implementation plan for the Caring for the Caregiver program.

## Methods

Caregiver and community engagement was undertaken in two phases. Phase 1 consisted of communication and collection of information from stakeholders to better understand needs and included an environmental scan of resources in the community for family caregiving. Phase 2 involved a collaboration with stakeholders to identify the goals for the Caring for the Caregiver Program. This process was guided by the principles of Asset Based Community Development (ABCD). [16] This project was submitted to the Institutional Review Board and deemed non-human subjects research.

### Communication and collection of information from stakeholders

The caregiver team (see author list) undertook an environmental scan to identify the community of interest, systematically developing a comprehensive list of community agencies and individuals who are involved with family caregivers of persons with dementia. This was performed through an internet search where we identified key advisory groups such as the Alzheimer’s Association and the local Area Agency on Aging Caregiver Program who further informed us about adult day centers, memory care homes, and geriatric clinics. Following this, four team members organized meetings and traveled to the different locations to meet with these groups to discuss our purpose and also to learn about their services for family caregivers, and potential gaps in services. Team members took time to develop a presence in the community, with involvement in community activities as an opportunity to begin building relationships and also to identify potential collaborators. We were invited to join community committees related to family caregiving and participated in community events, spoke at caregiver support groups, staffed booths at health fairs, and attended local educational events related to family caregiving. Two team members also met with family caregivers at support groups and local dementia clinics to discuss the adequacy of services and support, and potential gaps. We systematically documented the services offered by these agencies and also gaps in care from the perspectives of the stakeholders. This communication of information and relationship-building phase was key to our success in the next phase of the community and public involvement.

### Collaboration where stakeholders are partners

We held a community-academic forum on October 7, 2016 at UT Health San Antonio. We invited community agencies, family caregivers, clinicians, and university stakeholders including researchers to attend the forum. The session was opened by a nurse researcher who provided an update on the latest evidence on family caregiving for persons with dementia. This helped to build a shared understanding of family caregiving and set the stage for the small group discussion. Participants were assigned to one of 5 groups so that each group was comprised of family caregivers, people from community agencies, clinicians, and university stakeholders. Members of the Caring for the Caregiver team facilitated the small group discussions, which lasted for one hour. Three main categories of questions guided the small group discussion: i) What community resources have been most helpful for family caregivers of person with dementia? What makes it easy for you to provide care for your family members? ii) How can the San Antonio community better support family dementia caregivers by building on the existing strengths? What needs are not being met for caregivers in this city? iii) What would help you the most, right now, in caring for someone with dementia? Following small group discussion all participants reconvened for one hour to summarize and share the findings from the small groups. With the verbal permission of all group participants, both the small and large group discussions were audiotaped and transcribed for analysis, enhancing credibility of the data and the analysis. [17] Data were analyzed using thematic content analysis and triangulated with observations from our engagement activities and the current caregiving literature. Two reviewers worked independently and then compared findings to develop codes and synthesize these into themes. Themes were discussed by the team to reach a consensus. Credibility was also enhanced by the experience with family caregiving of several team members. [17]

## Results

### Communication and collection of information from stakeholders

We met with 15 community agencies in meetings ranging from 1 to 3 h to describe the goals of the Caring for the Caregiver program, to document services delivered by these community agencies, and to support potential future collaborations. There was a consensus across community agencies that there are important gaps in the provision and coordination of services and that there is an essential role for the Caring for the Caregiver program in practice, education, and research. Some examples include the need for an up-to-date registry of support services for family caregivers, need for a systematic approach to care for those with dementia and their families at the time of diagnosis, respite services, and the need for more community-based education about caregiving for families.

We also recruited 13 family caregivers, identified from a dementia clinic, to complete a 30-min survey by telephone. Caregivers represented the disease trajectory with those in the very early phase of caregiving to those who had been providing care for up to 10 years as well as two former caregivers who had cared for their dying spouses. The sample consisted of both spouses and adult children caring for relatives with dementia, was 67% female and 40% Hispanic. To increase the diversity of our sample, we also held a focus group with 15 caregivers living in an outlying rural community to identify their needs for support. This group was 90% Hispanic, 80% female, with similar diversity to above in kin relationship to their family member with dementia. Consistently caregivers described the challenges for their families resulting from providing care to a loved one with dementia. Caregiver’s needs included the difficulty of getting a diagnosis and knowing what to do at that time in regards to medical and legal power of attorney. The diagnosis phase was particularly challenging for caregivers from the rural setting who have less access to healthcare facilities and medical specialists. Caregivers also described their desire to meet with and learn from other caregivers in similar situations and the importance of peer support. As stated by one caregiver, *“I don’t need to go into a lengthy explanation when I am speaking to another caregiver…she knows and understands what I am going through without me needing to say very much at all.”* Finally, caregivers reported difficulty finding and applying for resources and assessing the quality of the care, suggesting the need for a “one-stop shopping” model. At the same time, caregivers want healthcare professionals to recognize their expertise in caring for their family members. Caregivers attending local support groups shared concerns with our team that were similar to the findings outlined above.

### Collaboration where stakeholders are partners

There were 53 participants who attended the community-academic forum. The distribution of participants at the forum was as follows: 28% members from community agencies, 25% family caregivers, 8% healthcare professionals, 22% from the university, and all members of the Caring for the Caregiver team (17%).

Participants listed a number of assets within the San Antonio community for family caregiving (see Appendix 1). In response to question 2 which explored the needs of family caregivers and how they can be better supported by building on the existing strengths, the following themes were identified: i) making the invisible visible; ii) you don’t know what you don’t know; iii) learning too late; and iv) anticipating and preparing for the future.

#### Making the invisible visible

Participants discussed the need for increased awareness of the role of family caregivers. One of the difficulties is that caregivers themselves do not routinely self-identify with this role, but rather perceive their caregiving activities as part of their normal role as spouses or children and therefore become invisible to the community as a family caregiver. Participants from community agencies stated that without awareness of family caregivers, they are not able to provide them with needed services. As stated by a community partner, *“One of the things that we’ve recognized is that many caregivers don’t recognize themselves as caregivers. So education, I think, is extremely important to get the word out.”* Furthermore, caregiving is not part of our traditional social conversation so caregivers do not typically discuss their needs for services or support. This phenomenon may in part be related to the stigma that continues to be associated with dementia but, nonetheless, it contributes to the difficulty caregivers experience in navigating and accessing available resources and support when needed. As one caregiver stated at the forum, “*You go into this and you’re like help! And I don’t know where – you feel alone. You don’t know what is out there, and then once you’re through it… people don’t talk about it. It shocked us how many friends we had that suddenly “Oh yeah, we did that with our mom.” Well why didn’t we know that?”*

#### You don’t know what you don’t know

Caregivers consistently described being provided with limited information and not knowing how or where to ask for help, *“you may not be at a place where you know even where to go to for help, and where to start”*. Community agencies described the challenges of identifying and engaging caregivers to make them aware of services as families may not be provided, at the time of diagnosis, with information about relevant community agencies such as the Alzheimer’s Association. Healthcare professionals described not knowing the resources available in the community which subsequently limited timely and appropriate referrals for caregivers. As one community agency member stated, *“And I think sometimes, they don't know what they don’t know.”* Family caregivers described diagnosis as a particularly difficult time, being in shock, and not knowing how to proceed. *“He was diagnosed…. We were not really told anything. He had a memory problem. And you’re just kind of thrown out there. “Now, go figure it out.””* Consistent across the groups was the importance of the time of diagnosis, the limited resources that are focused on supporting families during the diagnostic phase, but also the importance of connecting with caregivers at this time.

#### Learning too late

A lack of information, as highlighted by the theme above, was related to the perceived timeliness of information. Consistently it was stated that information seems to come *“at random”*. There is not a systematic way that information about resources and support is delivered. Caregivers describe learning too late of available support and services that would have changed their earlier decisions and potentially their caregiving situation. As one caregiver stated, *“my thought is that if somebody would have talked to me earlier.”* A provider who also was a family caregiver stated, “*I never appreciated how hard it was for families to find good quality home health care in this community until I had to do it myself. And I thought I knew how to do it and it was a nightmare.”* Participants discussed that one of the challenges of timely access to resources and support was that information is found in multiple different places and ways rather than through a streamlined system which would help to ensure that people receive timely information and support through-out the different phases of the disease and their caregiving trajectory. Participants emphasized the importance of better dissemination of the information about resources. As one community member stated, *“there are some resources out there, again, but without marketing them, people don’t know where to start… they’re so stressed that they’re just searching for anything that’s going to help save them from sinking…”.*

#### Anticipating and preparing for the future

Caregivers discussed not knowing that Alzheimer’s disease is a chronic, progressive illness, a common misperception among not only caregivers, but also the community at large. Caregivers described how they were unaware early on that Alzheimer’s disease is a terminal illness which limited their ability to plan and access resources to support them and their loved one through advanced stages of dementia. A participant from the community stated, *“this is another area that needs to be dealt with as well, helping the public to understand that sometimes or in all cases, there won’t be improvement.”* One caregiver stated, “*We were never told this was a terminal illness – we were not prepared to deal with the situation as it progressed.”* Another caregiver described finally getting hospice care the last 3 days of her husband’s life, *“I didn’t get hospice in early enough – didn’t know to look at this – it was a god-send when it happened.”* Healthcare professionals discussed the need for palliative care and earlier information about future care to help patients plan and also to support family caregivers. This includes advance care planning and advanced dementia-specific care.

## Discussion

The process of collaborating with community stakeholders offers great promise in establishing a relevant Caring for the Caregiver program. Engaging with stakeholders has led to an extensive network of community partners, culminating in the community-academic forum. This is, to our knowledge, the first time that relevant stakeholders in the area of family caregiving for persons with dementia have come together in our community to discuss how we can move the agenda forward to offer needed support for family caregivers. The mission statements of several societies for dementia include the importance of patient and public involvement in decisions that impact on the lives of people with dementia. [18, 19] There are recent publications reporting on the process of involving family caregivers and other community partners in identifying priorities for practice and research; however, the main focus has been on the care of the person with dementia rather than the family caregiver. [20–23]

Although there were differing viewpoints, having the common goal of improving care and support for family caregivers in our community united all stakeholders. All comments were recorded and experienced facilitators ensured that all participants were afforded the opportunity to express their perspectives. The themes identified in the discussion were broad and ranged from raising awareness about family caregivers to specific care issues. In particular, there was a focus on transitions, where caregivers may be more vulnerable to risks that may in turn affect their health [24, 25]. At the time of diagnosis with Alzheimer’s disease or other forms of dementia, the family caregiver is faced with new challenges in identifying as a caregiver, developing self-confidence in this role including the mastery of specific skills to manage the new situations, knowledge about relevant services, and connecting with an informal support network. [26] Participants in the forum identified this as important gap in education and support. Anticipating and preparing for the transition to advanced dementia care was also recognized as critical, with a need to build on the assets in the community related to advance care planning, specifically for caregivers of persons with dementia. There is a rising trend for people with dementia dying at home (13.9% in 1999 to 24.9% in 2014), [27] highlighting the need for caregiver support in this demanding role.

Using patient and public involvement, the James Lind Alliance identified priorities for research in dementia. [28] The top 10 priorities included care at the time of transitions and raising public awareness about dementia to create dementia friendly environments. These themes are consistent with findings identified through our caregiver and public involvement. There is an extensive evidence base for interventions for family caregivers of persons with dementia, yet few interventions have been implemented into practice. [9] There is an urgent need to extend partnerships with family caregivers and community stakeholders to better understand methodology for integrating best practice into routine care for caregivers. The data from our community engagement will not only direct our practice and education, but also inform future research.

One of the challenges in this process was the extensive time required to identify and meet with our stakeholders and to begin to build trusting relationships. We needed to be sensitive to their schedules, particularly with meeting family caregivers who often have limited time outside their caregiving role. Visibility is the key to relationship building so in addition to meeting with our stakeholders, we regularly attended community events, contributing when it was appropriate, and have taken roles on relevant committees to further the agenda of family caregivers for persons with dementia in this community. Although we have spent over one year building these relationships, the benefits of taking the time to establish meaningful relationships with the community has led to collaboration that is characterized by mutual capacity building, asset sharing, and submission to date of four co-designed funding applications (with community partners, including family caregivers, as peer researchers) that will further our knowledge about best practice to support family caregivers. These applications are a result of the mutual understanding that emerged from our partnerships.

There are limitations in our methods of collecting information from our stakeholders. Convenience sampling was used for family caregivers and as such, the information from these caregivers may represent those who are able to attend a focus group or take the time for a telephone interview and may not represent caregivers most in need of support. We are aware of the issue of personal bias in analyzing the qualitative data and how this may have influenced our findings. As we were a large team analyzing the data and debriefing, this may have protected against this potential bias. Finally, the time that we have taken to better understand our community and develop these relationships with our stakeholders may not be feasible for all groups. It is not certain, however, that the trusting partnerships that we have established, with ongoing support and collaboration, would have occurred without such extensive involvement.

### Next steps

We have provided feedback from our forum and other activities to our caregiver community stakeholders. Feedback from participants about the forum included the importance of these conversations where everyone is in the same room and planning together how our community can address the needs and provide support for family caregivers of person with dementia. They also described the benefit of the forum in connecting people to people, people to organizations, and creating bonds. Based on these data, we have established teams that include stakeholders from the community around projects that encompass practice, education, and research. Phase 1 is the development of a caregiver curriculum and website support. Included in this project is the development of a new diagnosis program, resource website, and skills training workshop, including simulation for caregivers. Phase 2 is the implementation of advance care planning in clinics and an advanced dementia care collaborative with community agency and caregiver stakeholders. Based on the success of our initial forum, we will make it an annual event where we can continue to collaborate with our stakeholders on mutually established goals.

## Conclusion

In developing the Caring for the Caregiver Program, we have used a process where caregivers have been given a voice in describing the issues of relevance in a program designed to address their well-being. In this project, we have been able to bring together a diverse group of stakeholders, reflecting the diversity in our community. We believe that the process of identifying and establishing relationships with our stakeholders will result in a sustainable program for family caregivers. Community engagement will also support the translation of interventions into support services to meet the needs of the growing population of family caregivers.

## Appendix 1

### Assets within the community

Adult Day Centers and Senior Centers.

Alzheimer’s Association.

Area Agency on Aging Caregiver Program.

Research Institutes at the university for aging.

Training classes for caregivers including Stress Busters Program and Powerful Tools for Caregivers.

Opportunities for respite care.

Support groups through-out the city, including bilingual support groups.

Veterans Administration Caregiver Program.

Inter-faith community and their support for family caregivers.

Grocery store and pharmacy home delivery.

mmLearn website for caregiver training videos that is located in the community.

Opportunities for assistance with resource management.
